# 2-[(*E*)-Meth­oxy­imino]-2-{2-[(2-methyl­phen­oxy)meth­yl]phen­yl}ethanoic acid

**DOI:** 10.1107/S1600536812030711

**Published:** 2012-07-10

**Authors:** Rajni Kant, Vivek K. Gupta, Kamini Kapoor, Chetan S. Shripanavar, Kaushik Banerjee

**Affiliations:** a X-ray Crystallography Laboratory, Post-Graduate Department of Physics and Electronics, University of Jammu, Jammu Tawi 180 006, India; b National Research Centre for Grapes, Pune 412 307, India

## Abstract

In the title compound, C_17_H_17_NO_4_, the dihedral angle between the two aromatic rings is 59.64 (5)°. The (meth­oxy­imino)­ethanoic acid fragment is nearly perpendicular to the attached benzene ring [dihedral angle = 81.07 (4)°]. In the crystal, pairs of O—H⋯O hydrogen bonds between carb­oxy groups link mol­ecules into inversion dimers. In addition, π–π stacking inter­actions between inversion-related benzene rings are observed [centroid–centroid distance = 3.702 (1) Å].

## Related literature

For the biological activities of kresoxim-methyl, see: Balba (2007 ▶); Cash & Cronan (2001 ▶); Ammermann *et al.* (2000 ▶). For a related structure, see: Chopra *et al.* (2004 ▶).
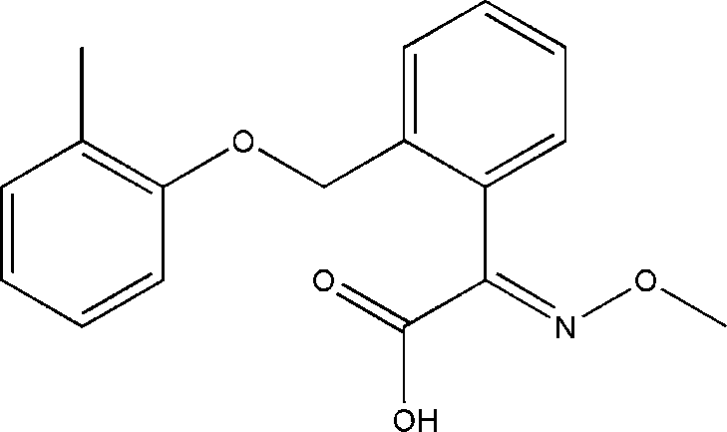


## Experimental

### 

#### Crystal data

C_17_H_17_NO_4_
*M* *_r_* = 299.32
Triclinic, 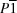

*a* = 7.8993 (3) Å
*b* = 8.5720 (3) Å
*c* = 12.6080 (5) Å
α = 88.013 (3)°
β = 82.270 (3)°
γ = 65.717 (4)°
*V* = 770.92 (5) Å^3^
*Z* = 2
Mo *K*α radiation
μ = 0.09 mm^−1^
*T* = 293 K
0.3 × 0.2 × 0.1 mm

#### Data collection

Oxford Diffraction Xcalibur Sapphire3 diffractometer
Absorption correction: multi-scan (*CrysAlis PRO*; Oxford Diffraction, 2010 ▶) *T*_min_ = 0.947, *T*_max_ = 1.000
18021 measured reflections
3016 independent reflections
2446 reflections with *I* > 2σ(*I*)
*R*_int_ = 0.031

#### Refinement

*R*[*F* ^2^ > 2σ(*F* ^2^)] = 0.037
*wR*(*F* ^2^) = 0.103
*S* = 1.05
3016 reflections
202 parameters
H-atom parameters constrained
Δρ_max_ = 0.21 e Å^−3^
Δρ_min_ = −0.14 e Å^−3^

### 

Data collection: *CrysAlis PRO* (Oxford Diffraction, 2010 ▶); cell refinement: *CrysAlis PRO*; data reduction: *CrysAlis PRO*; program(s) used to solve structure: *SHELXS97* (Sheldrick, 2008 ▶); program(s) used to refine structure: *SHELXL97* (Sheldrick, 2008 ▶); molecular graphics: *ORTEP-3* (Farrugia, 1997 ▶); software used to prepare material for publication: *PLATON* (Spek, 2009 ▶).

## Supplementary Material

Crystal structure: contains datablock(s) I, global. DOI: 10.1107/S1600536812030711/gk2509sup1.cif

Structure factors: contains datablock(s) I. DOI: 10.1107/S1600536812030711/gk2509Isup2.hkl

Supplementary material file. DOI: 10.1107/S1600536812030711/gk2509Isup3.cml

Additional supplementary materials:  crystallographic information; 3D view; checkCIF report

## Figures and Tables

**Table 1:** Hydrogen-bond geometry (Å, °)

*D*—H⋯*A*	*D*—H	H⋯*A*	*D*⋯*A*	*D*—H⋯*A*
O1—H1⋯O2^i^	0.82	1.82	2.640 (2)	176

Symmetry code: (i) 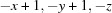.

**Fig. 1.**
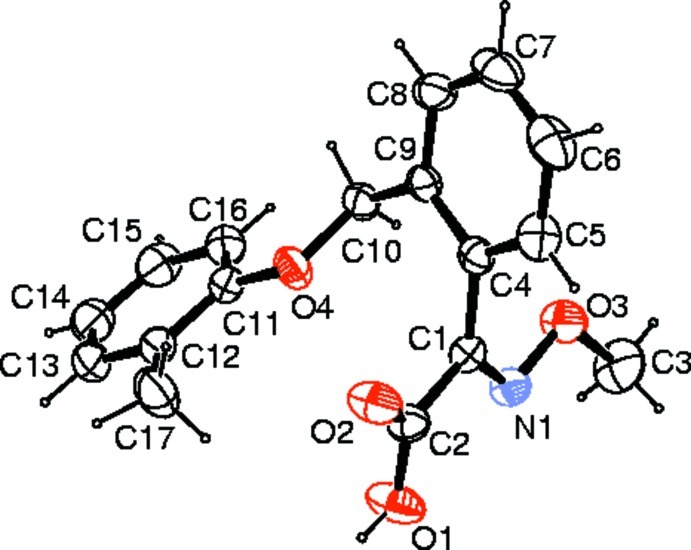
ORTEP view of the molecule with the atom-labeling scheme. The displacement ellipsoids are drawn at the 40% probability level. H atoms are shown as small spheres of arbitrary radii.

**Fig. 2.**
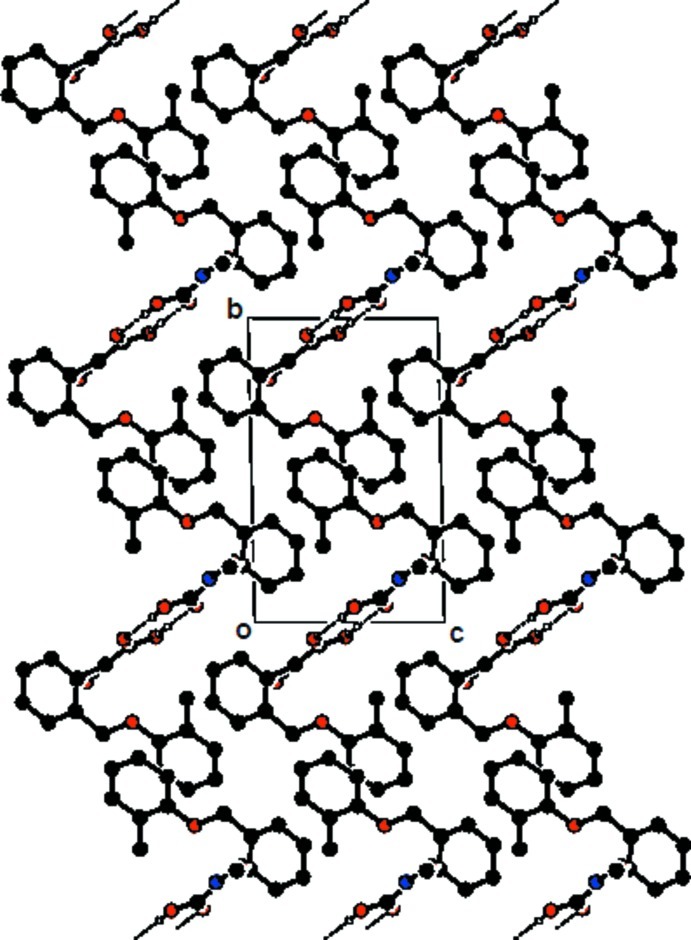
The packing arrangement of molecules viewed down the a axis. The dotted lines show intermolecular O—H···O hydrogen bonds.

## supplementary crystallographic information

### Comment

The title compound is the acid metabolite of kresoxim-methyl, which is a systemic fungicide of strobilurin group with broad spectrum bio-efficacy against various diseases (Balba, 2007; Cash & Cronan, 2001; Ammermann *et al.*, 2000) of economically important agricultural crops.

In (I), all bond lengths and angles are normal and correspond to those observed in the related structure (Chopra *et al.*, 2004). The dihedral angle between the the two aromatic rings is 59.64 (5)°. The (methoxyimino)ethanoic acid fragment is nearly perpendicular to the attached benzene ring [dihedral angle 81.07 (4)°]. In the crystal, O—H···O hydrogen bonds link pairs of molecules to form inversion dimers (Fig. 2). The crystal structure is further stabilized by π–π interactions between the benzene ring (C11—C16) of the molecule at (*x*, *y*, *z*) and the benzene ring of an inversion related molecule at (1 - *x*, 1 - *y*, 1 - *z*)[centroid separation = 3.702 (1) Å, interplanar spacing = 3.547Å and centroid shift = 1.05 Å].

### Experimental

Kresoxim-methyl (0.313 g, 0.001 mol) was dissolved in 5 ml acetone and to it 5 ml of 1 N NaOH solution was added. The reaction mixture was refluxed on a water bath at 343 K for 12 hrs, and then cooled. The compound was precipitated by neutralizing with 1 N HCl solution. The precipitated compound was dissolved in methanol and crystallized by the process of slow evaporation (m.p. 413 K).

### Refinement

All H atoms were positioned geometrically and were treated as riding on their parent C / O atoms, with O—H distance of 0.82 Å and C—H distances of 0.93–0.97 Å and with *U*_iso_(H) = 1.2*U*_eq_(C) or 1.5*U*_eq_(methyl C / O).

### Crystal data

**Table:**

C_17_H_17_NO_4_	*Z* = 2
*M**_r_* = 299.32	*F*(000) = 316
Triclinic, *P*1	*D*_x_ = 1.289 Mg m^−^^3^
Hall symbol: -P 1	Mo *K*α radiation, λ = 0.71073 Å
*a* = 7.8993 (3) Å	Cell parameters from 8697 reflections
*b* = 8.5720 (3) Å	θ = 4.0–29.0°
*c* = 12.6080 (5) Å	µ = 0.09 mm^−^^1^
α = 88.013 (3)°	*T* = 293 K
β = 82.270 (3)°	Block, colourless
γ = 65.717 (4)°	0.3 × 0.2 × 0.1 mm
*V* = 770.92 (5) Å^3^	

### Data collection

**Table:**

Oxford Diffraction Xcalibur Sapphire3 diffractometer	3016 independent reflections
Radiation source: fine-focus sealed tube	2446 reflections with *I* > 2σ(*I*)
Graphite monochromator	*R*_int_ = 0.031
Detector resolution: 16.1049 pixels mm^-1^	θ_max_ = 26.0°, θ_min_ = 4.0°
ω scan	*h* = −9→9
Absorption correction: multi-scan (*CrysAlis PRO*; Oxford Diffraction, 2010)	*k* = −10→10
*T*_min_ = 0.947, *T*_max_ = 1.000	*l* = −15→15
18021 measured reflections	

### Refinement

**Table:**

Refinement on *F*^2^	Secondary atom site location: difference Fourier map
Least-squares matrix: full	Hydrogen site location: inferred from neighbouring sites
*R*[*F*^2^ > 2σ(*F*^2^)] = 0.037	H-atom parameters constrained
*wR*(*F*^2^) = 0.103	*w* = 1/[σ^2^(*F*_o_^2^) + (0.0464*P*)^2^ + 0.1581*P*] where *P* = (*F*_o_^2^ + 2*F*_c_^2^)/3
*S* = 1.05	(Δ/σ)_max_ = 0.001
3016 reflections	Δρ_max_ = 0.21 e Å^−^^3^
202 parameters	Δρ_min_ = −0.14 e Å^−^^3^
0 restraints	Extinction correction: *SHELXL97* (Sheldrick, 2008), Fc^*^=kFc[1+0.001xFc^2^λ^3^/sin(2θ)]^-1/4^
Primary atom site location: structure-invariant direct methods	Extinction coefficient: 0.129 (7)

### Special details

**Table:**

Experimental. *CrysAlis PRO*, Oxford Diffraction Ltd., Version 1.171.34.40 (release 27–08-2010 CrysAlis171. NET) (compiled Aug 27 2010,11:50:40) Empirical absorption correction using spherical harmonics, implemented in SCALE3 ABSPACK scaling algorithm.
Geometry. All e.s.d.'s (except the e.s.d. in the dihedral angle between two l.s. planes) are estimated using the full covariance matrix. The cell e.s.d.'s are taken into account individually in the estimation of e.s.d.'s in distances, angles and torsion angles; correlations between e.s.d.'s in cell parameters are only used when they are defined by crystal symmetry. An approximate (isotropic) treatment of cell e.s.d.'s is used for estimating e.s.d.'s involving l.s. planes.
Refinement. Refinement of *F*^2^ against ALL reflections. The weighted *R*-factor *wR* and goodness of fit *S* are based on *F*^2^, conventional *R*-factors *R* are based on *F*, with *F* set to zero for negative *F*^2^. The threshold expression of *F*^2^ > σ(*F*^2^) is used only for calculating *R*-factors(gt) *etc*. and is not relevant to the choice of reflections for refinement. *R*-factors based on *F*^2^ are statistically about twice as large as those based on *F*, and *R*- factors based on ALL data will be even larger.

### Fractional atomic coordinates and isotropic or equivalent isotropic displacement parameters (Å^2^)

**Table:**

	*x*	*y*	*z*	*U*_iso_*/*U*_eq_	
N1	−0.04205 (15)	0.76501 (15)	0.14263 (9)	0.0411 (3)	
O1	0.26565 (15)	0.52466 (14)	0.04947 (10)	0.0627 (4)	
H1	0.3655	0.4611	0.0157	0.094*	
O2	0.41631 (14)	0.69280 (13)	0.05567 (9)	0.0545 (3)	
O3	−0.19671 (13)	0.89490 (14)	0.19809 (9)	0.0537 (3)	
O4	0.29418 (13)	0.65865 (12)	0.32961 (7)	0.0470 (3)	
C1	0.10574 (17)	0.79233 (16)	0.14043 (10)	0.0343 (3)	
C2	0.27728 (18)	0.66279 (17)	0.07790 (11)	0.0389 (3)	
C3	−0.3626 (2)	0.8742 (3)	0.18052 (16)	0.0714 (5)	
H3A	−0.3753	0.8841	0.1056	0.107*	
H3B	−0.4704	0.9612	0.2203	0.107*	
H3C	−0.3529	0.7634	0.2039	0.107*	
C4	0.11852 (16)	0.94464 (16)	0.18597 (10)	0.0343 (3)	
C5	0.1139 (2)	1.07741 (19)	0.11881 (13)	0.0481 (4)	
H5	0.1053	1.0687	0.0466	0.058*	
C6	0.1217 (2)	1.2226 (2)	0.15794 (16)	0.0593 (5)	
H6	0.1148	1.3126	0.1128	0.071*	
C7	0.1399 (2)	1.2330 (2)	0.26406 (16)	0.0589 (5)	
H7	0.1464	1.3299	0.2908	0.071*	
C8	0.14853 (19)	1.10001 (19)	0.33090 (13)	0.0489 (4)	
H8	0.1627	1.1077	0.4023	0.059*	
C9	0.13653 (16)	0.95487 (16)	0.29377 (11)	0.0364 (3)	
C10	0.14079 (19)	0.81417 (17)	0.36887 (11)	0.0418 (3)	
H10A	0.1558	0.8422	0.4399	0.050*	
H10B	0.0240	0.8006	0.3734	0.050*	
C11	0.33196 (19)	0.51830 (17)	0.39422 (11)	0.0400 (3)	
C12	0.4895 (2)	0.37100 (18)	0.35617 (12)	0.0447 (3)	
C13	0.5341 (2)	0.22787 (19)	0.41993 (14)	0.0551 (4)	
H13	0.6382	0.1285	0.3965	0.066*	
C14	0.4296 (3)	0.2281 (2)	0.51667 (15)	0.0596 (4)	
H14	0.4637	0.1305	0.5579	0.072*	
C15	0.2752 (2)	0.3729 (2)	0.55158 (14)	0.0568 (4)	
H15	0.2033	0.3736	0.6165	0.068*	
C16	0.2255 (2)	0.5189 (2)	0.49049 (12)	0.0486 (4)	
H16	0.1205	0.6172	0.5144	0.058*	
C17	0.6052 (2)	0.3689 (2)	0.25175 (15)	0.0647 (5)	
H17A	0.7198	0.2672	0.2458	0.097*	
H17B	0.6334	0.4679	0.2484	0.097*	
H17C	0.5369	0.3702	0.1940	0.097*	

### Atomic displacement parameters (Å^2^)

**Table:**

	*U*^11^	*U*^22^	*U*^33^	*U*^12^	*U*^13^	*U*^23^
N1	0.0358 (6)	0.0487 (7)	0.0405 (6)	−0.0196 (5)	−0.0022 (5)	−0.0041 (5)
O1	0.0494 (6)	0.0518 (6)	0.0893 (9)	−0.0285 (5)	0.0166 (6)	−0.0286 (6)
O2	0.0408 (6)	0.0539 (6)	0.0715 (7)	−0.0260 (5)	0.0106 (5)	−0.0200 (5)
O3	0.0315 (5)	0.0610 (7)	0.0665 (7)	−0.0183 (5)	0.0021 (5)	−0.0155 (5)
O4	0.0466 (6)	0.0382 (5)	0.0395 (5)	−0.0021 (4)	−0.0011 (4)	0.0007 (4)
C1	0.0342 (7)	0.0385 (7)	0.0324 (6)	−0.0170 (6)	−0.0055 (5)	0.0023 (5)
C2	0.0377 (7)	0.0420 (7)	0.0407 (7)	−0.0207 (6)	−0.0020 (6)	−0.0044 (6)
C3	0.0362 (8)	0.0977 (14)	0.0844 (13)	−0.0323 (9)	−0.0035 (8)	−0.0062 (11)
C4	0.0262 (6)	0.0339 (7)	0.0411 (7)	−0.0112 (5)	−0.0026 (5)	−0.0007 (5)
C5	0.0460 (8)	0.0499 (8)	0.0516 (9)	−0.0235 (7)	−0.0062 (6)	0.0095 (7)
C6	0.0512 (9)	0.0417 (8)	0.0876 (13)	−0.0241 (7)	−0.0038 (9)	0.0121 (8)
C7	0.0440 (8)	0.0402 (8)	0.0953 (14)	−0.0206 (7)	−0.0037 (8)	−0.0142 (8)
C8	0.0375 (7)	0.0466 (8)	0.0590 (9)	−0.0127 (6)	−0.0045 (6)	−0.0178 (7)
C9	0.0252 (6)	0.0352 (7)	0.0426 (7)	−0.0063 (5)	−0.0023 (5)	−0.0067 (5)
C10	0.0391 (7)	0.0404 (7)	0.0357 (7)	−0.0061 (6)	−0.0033 (6)	−0.0044 (6)
C11	0.0402 (7)	0.0380 (7)	0.0421 (8)	−0.0139 (6)	−0.0136 (6)	0.0015 (6)
C12	0.0412 (7)	0.0398 (7)	0.0526 (8)	−0.0133 (6)	−0.0149 (6)	−0.0030 (6)
C13	0.0567 (9)	0.0359 (8)	0.0712 (11)	−0.0129 (7)	−0.0236 (8)	−0.0008 (7)
C14	0.0768 (12)	0.0452 (9)	0.0666 (11)	−0.0306 (9)	−0.0266 (9)	0.0139 (8)
C15	0.0678 (11)	0.0583 (10)	0.0534 (9)	−0.0341 (9)	−0.0124 (8)	0.0092 (8)
C16	0.0478 (8)	0.0471 (8)	0.0485 (9)	−0.0165 (7)	−0.0082 (7)	0.0018 (7)
C17	0.0535 (10)	0.0515 (10)	0.0675 (11)	−0.0022 (8)	0.0010 (8)	−0.0043 (8)

### Geometric parameters (Å, º)

**Table:**

N1—C1	1.2784 (16)	C7—H7	0.9300
N1—O3	1.3880 (14)	C8—C9	1.3875 (19)
O1—C2	1.2910 (16)	C8—H8	0.9300
O1—H1	0.8200	C9—C10	1.4994 (19)
O2—C2	1.2228 (16)	C10—H10A	0.9700
O3—C3	1.4391 (18)	C10—H10B	0.9700
O4—C11	1.3791 (16)	C11—C16	1.379 (2)
O4—C10	1.4305 (15)	C11—C12	1.4006 (19)
C1—C4	1.4901 (17)	C12—C13	1.388 (2)
C1—C2	1.4921 (18)	C12—C17	1.495 (2)
C3—H3A	0.9600	C13—C14	1.378 (2)
C3—H3B	0.9600	C13—H13	0.9300
C3—H3C	0.9600	C14—C15	1.368 (2)
C4—C5	1.3859 (19)	C14—H14	0.9300
C4—C9	1.3948 (18)	C15—C16	1.387 (2)
C5—C6	1.383 (2)	C15—H15	0.9300
C5—H5	0.9300	C16—H16	0.9300
C6—C7	1.374 (3)	C17—H17A	0.9600
C6—H6	0.9300	C17—H17B	0.9600
C7—C8	1.378 (2)	C17—H17C	0.9600
			
C1—N1—O3	111.46 (11)	C8—C9—C10	120.26 (13)
C2—O1—H1	109.5	C4—C9—C10	121.31 (11)
N1—O3—C3	108.63 (11)	O4—C10—C9	108.75 (10)
C11—O4—C10	116.98 (10)	O4—C10—H10A	109.9
N1—C1—C4	126.66 (12)	C9—C10—H10A	109.9
N1—C1—C2	115.03 (11)	O4—C10—H10B	109.9
C4—C1—C2	118.13 (10)	C9—C10—H10B	109.9
O2—C2—O1	124.35 (12)	H10A—C10—H10B	108.3
O2—C2—C1	119.61 (11)	O4—C11—C16	123.67 (12)
O1—C2—C1	116.04 (11)	O4—C11—C12	115.46 (12)
O3—C3—H3A	109.5	C16—C11—C12	120.87 (13)
O3—C3—H3B	109.5	C13—C12—C11	117.19 (14)
H3A—C3—H3B	109.5	C13—C12—C17	121.54 (14)
O3—C3—H3C	109.5	C11—C12—C17	121.27 (13)
H3A—C3—H3C	109.5	C14—C13—C12	122.24 (15)
H3B—C3—H3C	109.5	C14—C13—H13	118.9
C5—C4—C9	119.87 (12)	C12—C13—H13	118.9
C5—C4—C1	118.75 (12)	C15—C14—C13	119.49 (15)
C9—C4—C1	121.37 (11)	C15—C14—H14	120.3
C6—C5—C4	120.78 (15)	C13—C14—H14	120.3
C6—C5—H5	119.6	C14—C15—C16	120.20 (16)
C4—C5—H5	119.6	C14—C15—H15	119.9
C7—C6—C5	119.48 (15)	C16—C15—H15	119.9
C7—C6—H6	120.3	C11—C16—C15	120.01 (14)
C5—C6—H6	120.3	C11—C16—H16	120.0
C6—C7—C8	120.08 (14)	C15—C16—H16	120.0
C6—C7—H7	120.0	C12—C17—H17A	109.5
C8—C7—H7	120.0	C12—C17—H17B	109.5
C7—C8—C9	121.32 (15)	H17A—C17—H17B	109.5
C7—C8—H8	119.3	C12—C17—H17C	109.5
C9—C8—H8	119.3	H17A—C17—H17C	109.5
C8—C9—C4	118.43 (13)	H17B—C17—H17C	109.5
			
C1—N1—O3—C3	169.20 (13)	C1—C4—C9—C8	179.72 (11)
O3—N1—C1—C4	−2.39 (18)	C5—C4—C9—C10	179.60 (12)
O3—N1—C1—C2	−177.26 (11)	C1—C4—C9—C10	−0.97 (18)
N1—C1—C2—O2	167.44 (13)	C11—O4—C10—C9	173.59 (11)
C4—C1—C2—O2	−7.90 (19)	C8—C9—C10—O4	−121.94 (13)
N1—C1—C2—O1	−11.62 (19)	C4—C9—C10—O4	58.76 (15)
C4—C1—C2—O1	173.04 (12)	C10—O4—C11—C16	3.11 (19)
N1—C1—C4—C5	−96.47 (16)	C10—O4—C11—C12	−176.49 (11)
C2—C1—C4—C5	78.27 (16)	O4—C11—C12—C13	178.86 (12)
N1—C1—C4—C9	84.09 (17)	C16—C11—C12—C13	−0.8 (2)
C2—C1—C4—C9	−101.17 (14)	O4—C11—C12—C17	−0.6 (2)
C9—C4—C5—C6	−1.8 (2)	C16—C11—C12—C17	179.77 (14)
C1—C4—C5—C6	178.77 (13)	C11—C12—C13—C14	0.1 (2)
C4—C5—C6—C7	1.9 (2)	C17—C12—C13—C14	179.57 (15)
C5—C6—C7—C8	−0.6 (2)	C12—C13—C14—C15	0.6 (2)
C6—C7—C8—C9	−0.9 (2)	C13—C14—C15—C16	−0.6 (2)
C7—C8—C9—C4	1.1 (2)	O4—C11—C16—C15	−178.85 (13)
C7—C8—C9—C10	−178.26 (13)	C12—C11—C16—C15	0.7 (2)
C5—C4—C9—C8	0.29 (18)	C14—C15—C16—C11	0.0 (2)

### Hydrogen-bond geometry (Å, º)

**Table:**

*D*—H···*A*	*D*—H	H···*A*	*D*···*A*	*D*—H···*A*
O1—H1···O2^i^	0.82	1.82	2.640 (2)	176

Symmetry code: (i) −*x*+1, −*y*+1, −*z*.
